# Supplementary material for: Lack of sexual dimorphism in a mouse model of isoproterenol-induced cardiac dysfunction
Source: PLoS One. 2020 Jul 9;15(7):e0232507. doi: 10.1371/journal.pone.0232507 (PMC7347208; doi:10.1371/journal.pone.0232507)
Supplement: S1 Table — (DOCX) [file pone.0232507.s001.docx]

**Supplementary Table 1. Primer sequences used in this study**

| Gene | Forward Primer | Reverse Primer |
| --- | --- | --- |
| ANP | 5′-GGA GCC TAC GAA GAT CCA GC-3′ | 5′-TCC AAT CCT GTC AAT CCT ACC C-3′ |
| BNP | 5′-AGT CCT TCG GTC TCA AGG CA-3′ | 5′-CCG ATC CGG TCT ATC TTG TGC-3′ |
| TGF beta-1 | 5'-CTC TTG AGT CCC TCG CAT CC-3' | 5'-GGT CTC CCA AGG AAA GGT AGG-3' |
| Beta-actin | 5′- TAT TGG CAA CGA GCG GTT CC-3′ | 5′-GGC ATA GAG GTC TTT ACG GAT GTC-3′ |
