# Supplementary material for: Lack of sexual dimorphism in a mouse model of isoproterenol-induced cardiac dysfunction
Source: PLoS One. 2020 Jul 9;15(7):e0232507. doi: 10.1371/journal.pone.0232507 (PMC7347208; doi:10.1371/journal.pone.0232507)
Supplement: S2 Table — (DOCX) [file pone.0232507.s002.docx]

**Supplementary Table 2.** Two-way ANOVA (Repeated Measures) analysis for echocardiographic parameters after acute isoproterenol administration to male and female C57Bl/6NCrl mice. This table shows the P values for acute isoproterenol effect, sex effect, and the interaction between isoproterenol and sex. P<0.05 is considered statistically significant and written in bold.

|  | **Acute**  **Isoproterenol effect** | **Sex effect** | **Interaction between isoproterenol and sex** |
| --- | --- | --- | --- |
| Ejection fraction | **<0.0001** | 0.4 | 0.84 |
| Fractional shortening | **<0.0001** | 0.24 | 0.79 |
| LV end systolic volume | **<0.0001** | 0.78 | 0.65 |
| LV end diastolic volume | **0.0004** | 0.12 | 0.27 |
| Cardiac output | **0.009** | **0.02** | 0.65 |
| Heart rate | **0.0003** | 0.59 | 0.22 |
|  | | | |
