# Supplementary material for: Lack of sexual dimorphism in a mouse model of isoproterenol-induced cardiac dysfunction
Source: PLoS One. 2020 Jul 9;15(7):e0232507. doi: 10.1371/journal.pone.0232507 (PMC7347208; doi:10.1371/journal.pone.0232507)
Supplement: S3 Table — (DOCX) [file pone.0232507.s003.docx]

**Supplementary Table 3.** Two-way ANOVA analysis for echocardiographic parameters after prolonged isoproterenol administration to male and female C57Bl/6NCrl mice. This table shows the P values for chronic isoproterenol effect, sex effect, and the interaction between isoproterenol and sex. P<0.05 is considered statistically significant and written in bold.

|  | **Prolonged**  **Isoproterenol effect** | **Sex effect** | **Interaction between isoproterenol and sex** |
| --- | --- | --- | --- |
| Ejection fraction | **<0.0001** | 0.88 | 0.64 |
| Fractional shortening | **<0.0001** | 0.70 | 0.61 |
| LV end systolic volume | **<0.0001** | 0.52 | 0.46 |
| LV end diastolic volume | **0.005** | 0.56 | 0.40 |
| Cardiac output | **0.001** | 0.63 | 0.73 |
| LV mass | **0.0036** | **<0.0001** | 0.8297 |
| Heart weight/tibial length | **<0.0001** | **<0.0001** | 0.22 |
