# Supplementary material for: Lack of sexual dimorphism in a mouse model of isoproterenol-induced cardiac dysfunction
Source: PLoS One. 2020 Jul 9;15(7):e0232507. doi: 10.1371/journal.pone.0232507 (PMC7347208; doi:10.1371/journal.pone.0232507)
Supplement: S4 Table — (DOCX) [file pone.0232507.s004.docx]

**Supplementary Table 4.** Two-way ANOVA analysis for hypertrophic and fibrotic markers expression after prolonged isoproterenol administration to male and female C57Bl/6NCrl mice. This table shows the P values for chronic isoproterenol effect, sex effect, and the interaction between isoproterenol and sex. P<0.05 is considered statistically significant and written in bold.

|  | **Prolonged**  **Isoproterenol effect** | **Sex effect** | **Interaction between isoproterenol and sex** |
| --- | --- | --- | --- |
| ANP | **0.02** | 0.65 | 0.98 |
| BNP | **0.005** | 0.33 | 0.77 |
| TGF-beta1 | **<0.0001** | 0.79 | 0.28 |
