# Supplementary material for: Lack of sexual dimorphism in a mouse model of isoproterenol-induced cardiac dysfunction
Source: PLoS One. 2020 Jul 9;15(7):e0232507. doi: 10.1371/journal.pone.0232507 (PMC7347208; doi:10.1371/journal.pone.0232507)
Supplement: S5 Table — (DOCX) [file pone.0232507.s005.docx]

**Supplementary Table 5.** Two-way ANOVA (Repeated Measures) analysis for echocardiographic parameters after prolonged isoproterenol administration to castrated and sham-operated male C57Bl/6NCrl mice. This table shows the P values for chronic isoproterenol effect, castration effect, and the interaction between isoproterenol and castration. P<0.05 is considered statistically significant and written in bold.

|  | **Chronic**  **Isoproterenol effect** | **Castration**  **effect** | **Interaction between isoproterenol and Castration** |
| --- | --- | --- | --- |
| Ejection fraction | **0.002** | 0.47 | 0.43 |
| Fractional shortening | **0.001** | 0.45 | 0.36 |
| LV end systolic volume | **0.003** | 0.59 | 0.55 |
| LV end diastolic volume | **0.005** | 0.7 | 0.58 |
| Cardiac output | **0.007** | 0.47 | 0.8 |
| LV mass | **0.0005** | 0.8992 | 0.2017 |
|  | | | |
