# Supplementary material for: Lack of sexual dimorphism in a mouse model of isoproterenol-induced cardiac dysfunction
Source: PLoS One. 2020 Jul 9;15(7):e0232507. doi: 10.1371/journal.pone.0232507 (PMC7347208; doi:10.1371/journal.pone.0232507)
Supplement: S6 Table — (DOCX) [file pone.0232507.s006.docx]

**Supplementary Table *6*.** Two-way ANOVA (Repeated Measures) analysis for echocardiographic parameters after prolonged isoproterenol administration to ovariectomized and sham-operated female C57Bl/6NCrl mice. This table shows the P values for chronic isoproterenol effect, ovariectomy effect, and the interaction between isoproterenol and ovariectomy. P<0.05 is considered statistically significant and written in bold.

|  | **Prolonged**  **Isoproterenol effect** | **Ovariectomy**  **effect** | **Interaction between isoproterenol and Ovariectomy** |
| --- | --- | --- | --- |
| Ejection fraction | **0.001** | 0.82 | 0.16 |
| Fractional shortening | **0.0008** | 0.96 | 0.15 |
| LV end systolic volume | **0.0004** | 0.71 | 0.36 |
| LV end diastolic volume | **<0.0001** | 0.63 | 0.2 |
| Cardiac output | 0.37 | 0.09 | **0.03** |
| LV mass | **0.0063** | 0.1522 | 0.9664 |
|  | | | |
