# Supplementary material for: Lack of sexual dimorphism in a mouse model of isoproterenol-induced cardiac dysfunction
Source: PLoS One. 2020 Jul 9;15(7):e0232507. doi: 10.1371/journal.pone.0232507 (PMC7347208; doi:10.1371/journal.pone.0232507)
Supplement: Attachment — Submitted filename: Zordoky_PlosOne_Carlson_Review.docx [file pone.0232507.s007.docx]

# 1 Original Submission

## 1.1 Recommendation: Minor Revision

# 2 Comments to Author:

Ms. Ref. No.: PONE-D-20-10894

Title: Isoproterenol-induced Cardiac Dysfunction in Male and Female C57Bl/6 Mice

Grant, Abdelgawad, Lewis, Seelig, and Zordoky

Overview and general recommendation:

Sex differences in cardiovascular disease has been observed in clinical medicine, as well as within various animal models of cardiac disease. The exact manifestation of these differences varies considerably between species and models. This study investigated the sex differences in a model of excessive catecholamine-induced cardiovascular disease disease by administration of moderate (10 mg/kg) dose isoproterenol subcutaneously to male and female mice. Additionally, the role of sex hormones in this model was further investigated by use of gonadectomized male and female mice. Echocardiographic parameters, histopathology of myocardial tissue, and gene expression of fibrotic markers were assessed.

Overall, this paper is well-written and clear, and the conclusions drawn appear appropriate based on the data provided. The authors discuss the relation between their findings and a multitude of other published studies investigating the role of sex in models of cardiovascular disease in laboratory rodents. The authors are honest about the shortcomings of the second arm of the study regarding gonadectomized animals and do not seek to draw substantial conclusions from this data.

## 2.1 Broad Comments

Methods: It would be helpful to clarify the amount of time that has passed from gonadectomy to use in this study for the altered mice. This will alleviate any concerns regarding an incomplete washout period, where residual sex hormones may still be circulating.

Discussion: It is my opinion that a brief discussion of the variables that were of significant difference between sexes (CO in the acute phase, LV mass and HW/TL ratio in the chronic phase), and why these differences still allow the author to draw the conclusion that this model lacks significant sex-related differences, would strengthen the authors position. These items are currently left out of the discussion, and may leave readers wondering if these factors are being intentionally ignored.

## 2.2 Specific Comments

Line 63: It would be helpful to characterize the class of pharmacologic agent.

Line 64: Recommend clarifying that this is used to induce cardiac pathologies in laboratory animal models.

Line 81: Recommend providing the exact strain name at least in the Methods section (C57BL/6NCrl)

Line 106: Specify the type of trichrome stain

Line 210: Recommend adding “animals” after the last “sham” of this sentence.

Line 221: Either replace “ISO” with isoproterenol or introduce this abbreviation earlier

Line 246-248: The wording of this sentence is such that it seems to say that you have found sex-related differences, when your conclusion is in fact the opposite. Instead of determined, consider “investigated”, or similar terminology to clarify that you were looking in to the possibility of these differences, not that they were determined.

Figure 6: Suggest matching the language between the legend (CAST) and figure itself (CASTRATED)
