# Supplementary material for: Lack of sexual dimorphism in a mouse model of isoproterenol-induced cardiac dysfunction
Source: PLoS One. 2020 Jul 9;15(7):e0232507. doi: 10.1371/journal.pone.0232507 (PMC7347208; doi:10.1371/journal.pone.0232507)
Supplement: Attachment — Submitted filename: PLOS One Response to Reviewers_R2.docx [file pone.0232507.s009.docx]

**Response to Reviewers:**

We would like to thank the editor and reviewers for their time and effort in reviewing our first submission and for providing their valuable comments which have improved the quality and clarity of the manuscript. Please find the following detailed response to the raised concerns.

**Reviewer #1: Thanks for your valuable responses that for sure improved the understanding and message of your study.**

**Response:** We are truly appreciative to the reviewer for their comments.

**Reviewer #2:
Overview and general recommendation:
The authors have thoroughly addressed this reviewer’s concerns, and the paper is well written and draws valid conclusions.**

**Response:** We are truly appreciative to the reviewer for their comments.

**SPECIFIC COMMENTS
Line 131 – 133: The scale for histopathology currently reads, “0, absent; 1, minimal inflammation or fibrosis; 2, mild minimal inflammation or fibrosis; 3, moderate minimal inflammation or fibrosis; and 4, marked minimal inflammation or fibrosis.”. Is the word “minimal” in 2, 3, and 4 an error?**

**Response:** We would like to thank the reviewer for catching this typographical error. The word “minimal” in 2, 3, and 4 was an error and omitted in the revised submission.

Lines 130 – 133 now read: “*Inflammation and fibrosis were assessed as follows: 0, absent; 1, minimal inflammation or fibrosis; 2, mild inflammation or fibrosis; 3, moderate inflammation or fibrosis; and 4, marked inflammation or fibrosis*”.
